# Setting a Nigeria national malaria operational research agenda: the process

**DOI:** 10.1186/s12913-018-3224-5

**Published:** 2018-06-18

**Authors:** Olufemi Ajumobi, Perpetua Uhomoibhi, Pamela Onyiah, Obafemi Babalola, Salami Sharafadeen, Maduka D. Ughasoro, Al-Mukhtar Y. Adamu, Oluwaseun Odeyinka, Taiwo Orimogunje, Ibrahim Maikore, Emmanuel Shekarau, Akintayo Ogunwale, Rotimi Afolabi, Sylvester Udeh, Akpuh Ndubuisi, Ntiense Umoette, Patrick Nguku, IkeOluwapo O. Ajayi

**Affiliations:** 1 Nigeria Field Epidemiology and Laboratory Training Programme, Abuja, Nigeria; 2 African Field Epidemiology Network, Abuja, Nigeria; 3 National Malaria Elimination Programme, Abuja, Nigeria; 4 Department of Paediatrics, University of Nigeria Enugu Campus, Enugu, Nigeria; 5 Department of Medical Microbiology and Parasitology, Faculty of Clinical Sciences, Bayero University, Kano, Nigeria; 6 Department of Epidemiology and Medical Statistics, Faculty of Public Health, College of Medicine, University of Ibadan, Ibadan, Nigeria; 7 Department of Health Promotion and Education, Faculty of Public Health, College of Medicine, University of Ibadan, Ibadan, Nigeria; 8 Department of General Studies, Oyo State College of Agriculture and Technology, Igboora, Oyo State Nigeria

**Keywords:** Malaria, Operational research, Agenda setting processes, Systematic approach, Nigeria

## Abstract

**Background:**

Employing malaria operational research (MOR) findings in planning national malaria control programmes is gaining increased attention. The malaria control foci are diverse, resources are limited; therefore, agreeing on priority areas is critical. Hitherto, the process of prioritising MOR questions in Nigeria has been limited to few stakeholders. In support of the National Malaria Elimination Programme’s (NMEP) effort at setting a MOR agenda, the Nigeria Field Epidemiology and Laboratory Training Programme (NFELTP) in collaboration with NMEP conducted preliminary exploratory study to identify key malaria research gaps and needs, and provide data to inform setting a robust national MOR agenda. The process of generating data is presented in this paper.

**Methods:**

A twelve-member task-team comprising NFELTP, university researchers and NMEP officers was commissioned. Following an inaugural meeting the task-team developed a framework of activities and held five planning meetings, conducted five-week online and self-administered paper-based surveys, key informant interview (KII), two-day desk review workshop, seven-day qualitative data analysis, ten-day result and five-day report writing workshops. Paired group members conducted the interviews across six geopolitical zones of Nigeria. Abridged study report was used for a two-day MOR setting agenda stakeholders’ workshop.

**Results:**

A structured framework, study protocol and data collection instruments were developed and submitted for ethical approval. The instruments included survey questionnaire for detailed information on researchers and other stakeholders’ experience with MOR, the gaps and needs in thematic MOR areas; KII and Delphi guides. After an initial scoping review, primary data were collected from purposively selected survey participants using mixed methods: - online survey (*n* = 100), self-administered paper-based survey (*n* = 85), KII (*n* = 40), desk review workshop (*n* = 22) and Delphi interviews (n = 8). Comprehensive lists of research gaps/bottlenecks and needs were generated for each thematic area in malaria control. These were used at a two-day national MOR setting stakeholder workshop (*n* = 54) to guide the development of national MOR agenda document.

**Conclusions:**

A systematic approach involving broad stakeholder engagement provided data and evidence-based information for development of a robust national MOR agenda. The processes involved are recommended for use in malaria endemic settings.

**Electronic supplementary material:**

The online version of this article (10.1186/s12913-018-3224-5) contains supplementary material, which is available to authorized users.

## Background

Globally, millions of deaths attributable to malaria are still being recorded. The disease constitutes a huge epidemiologic burden in Africa and continues to cripple the economic development in the region evidenced by most deaths compared to other regions of the world [1]. In Nigeria, the disease is responsible for 60% of outpatient visits to health facilities, 30% childhood death, and 11% maternal death [2]. It is estimated that Nigeria account for 29 and 26% of global malaria morbidity and mortality respectively [1]. The financial loss due to malaria annually is estimated to be about 132 billion Naira in form of treatment costs, prevention and loss of man-hours among other expenses; yet, it is a treatable and completely evitable disease [2].

The huge investment on malaria control for over a decade and half, has led to availability of new and efficient tools and significant reduction in morbidity and mortality globally, but the coverage targets are not yet met and implementation of strategies still fall short of expectation [1]. Harnessing innovation and expanding research is the first of the supporting elements of the three main pillars of the World Health Organisation’s Global Technical Strategy for malaria [3], without which global malaria control and elimination cannot be achieved. Hence, the need for malaria operational research to better understand the challenges to successful implementation of interventions and test newer effective strategy. Operational research is crucial to ascertaining the effectiveness and efficiency of current interventions in different settings as well as maximizing effect of deployment of new and innovative interventions [4].

The goal of the current Nigeria National Malaria Strategic Plan (NMSP) is to reduce morbidity to pre-elimination levels by 2020 and mortality to zero. Prioritisation of malaria operational research (MOR) for effective malaria control and eventual elimination is a clearly outlined strategy in the NMSP [2]. The National Malaria Elimination Programme (NMEP) supported by other malaria stakeholders and partners previously held workshops on improving MOR in Nigeria in 2010, 2012 and 2013 (United Kingdom Department for International Development-funded Support to National Malaria Programme [SuNMaP]); and a country dialogue and workshop on MOR in 2014 (funded by Roll Back Malaria [RBM] Partnership through West African RBM Network). Fallout from this workshop was the production of a list of harmonised malaria OR questions prioritised by relevance and thematic areas of NMEP for the lifespan of NMSP.

However, NMEP has not had the opportunity to take stock on progress made so far with regards to uptake of the prioritised research questions by relevant stakeholders. There might be emergence of new MOR questions which need to be addressed. Additionally, hitherto, the process of prioritising MOR questions in Nigeria has been limited to few stakeholders. There is therefore a need to revise the list of prioritised OR questions in the light of current realities, research questions emanating from the recent Nigeria Malaria Indicator Survey (NMIS, 2015) and reflect new and emerging questions. In addition, other research gaps and needs are desired to be identified and afterwards used to develop a national MOR agenda [5]. In support of the NMEP’s effort at setting a MOR agenda that aligns to the current NMSP, the Nigeria Field Epidemiology and Laboratory Training Programme (NFELTP) commissioned a task-team to conduct preliminary exploratory study using a more encompassing stakeholder participation to identify specific MOR gaps for developing a robust 2016–2020 national MOR agenda. The agenda will inform research efforts towards reduction of malaria burden to pre-elimination levels. This paper describes the processes for generating the MOR gaps, bottlenecks and needs used to develop and prioritised MOR questions for Nigeria using a consultative design.

## Methods

### Study scope

As part of the process we considered various modalities useful in collection of data including accessing policymakers, documents reviews, literature and desk reviews as well as representation of the geopolitical zones.

### Scoping review

The scoping review entailed review of literature and documents on malaria research in and outside this country and prior MOR agenda setting in Nigeria. In addition, systematic search of papers published on malaria in past ten years was carried out and extraction of contact details of authors/researchers in Nigeria and international researchers who have worked on malaria in Nigeria was done for the purpose of possible engagement in online or self-administered paper-based survey.

First, online literature was surveyed to have an understanding on how other countries carried out the process of setting operational research agenda. The search was conducted using Pubmed, Web of Science, Google Scholar and Medline. Recent publications from 2010 to 2016 were included. The keywords’ combination used for the search were: malaria, agenda setting, operation research and priorities. This revealed six articles, but no African country except Tanzania has a documented evidence for conducting MOR agenda setting using a structured approach [6]. Moreover, three relevant articles and one document were found by web search and these guided consultation processes for identifying research gaps, needs and priorities [6–9].

Weiner summarised activities involved in assessing and creating implementation readiness as well as tools and measures for assessing readiness and strategies to increase organisational readiness for change [7]. This informed the need for appropriate stakeholder engagement (including formation of a planning committee with wide stakeholder representation) and resulted in leadership role of NMEP in setting the Nigeria national MOR.

The review by Alonso et al. described malaria research agenda setting for malaria eradication [8]. This article recognised the need for redefining key knowledge gaps, developing appropriate strategies and presence of political will to achieve global malaria eradication. This further reinforced the need for a consultative process, political commitment and formation of MOR agenda setting preliminary survey task-team to ensure a scientific process is followed through in setting the Nigeria national MOR.

**Fig. 1:**
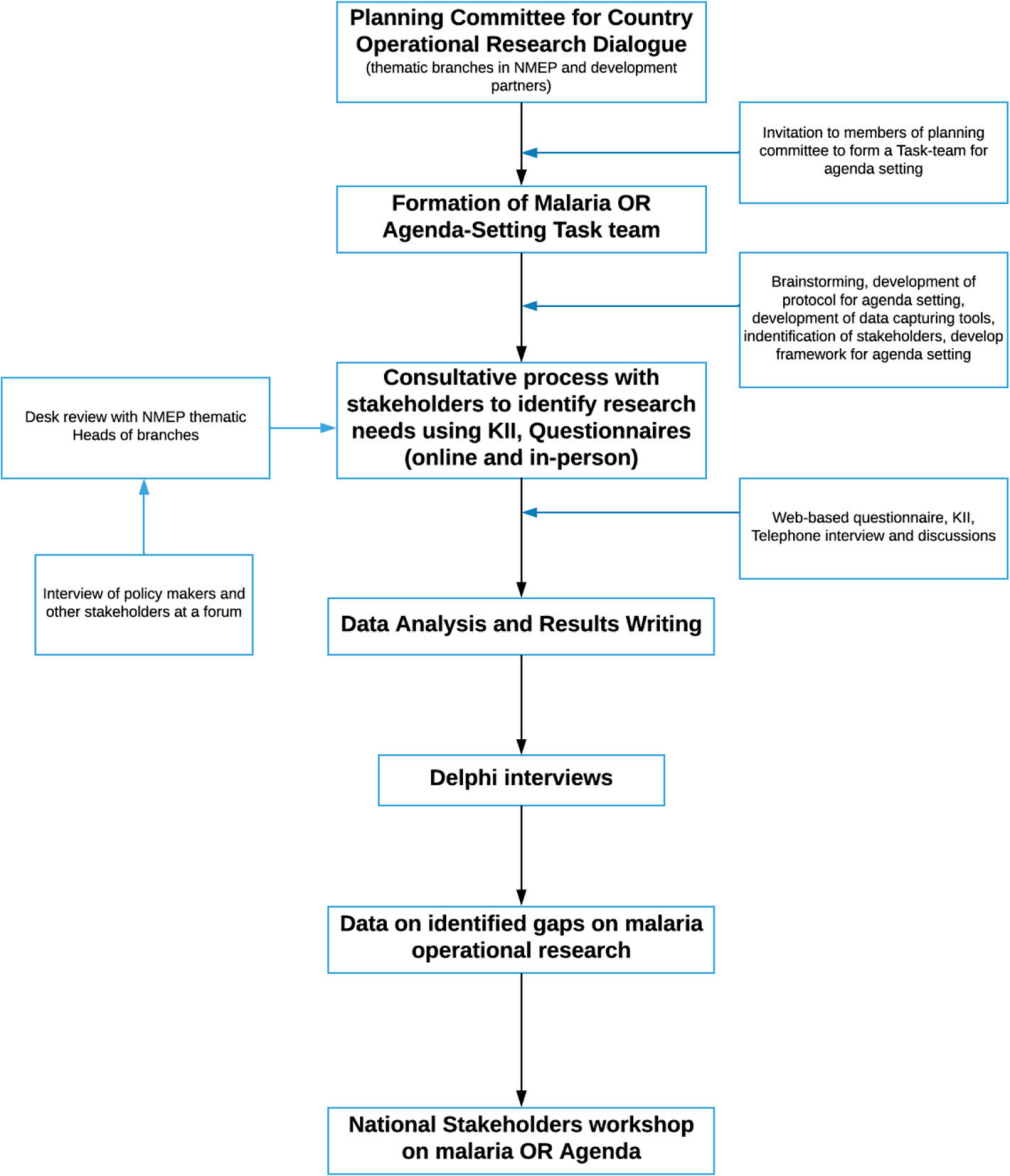
Framework for structured approach to generating gaps for malaria operational research agenda

The third article by Woodward et al. described the process of research agenda setting in fragile and conflict-affected ‘FCAS’ areas which have weak health systems and these make implementation of well-known health strategies and technologies challenging [9]. The processes described in this article guided the development of framework for structured approach to generating gaps for malaria operational research agenda in Nigeria (Fig. 1). Additionally, the questionnaire for our study was adapted from that used by Woodward et al. [9].

The fourth publication highlighted gaps and bottlenecks that limit access to and delivery of technologies for malaria, tuberculosis and neglected tropical diseases in Tanzania, the major causes and reasons, and programmatic responses [6]. This informed desk review/situational analysis workshop with NMEP thematic heads of branches and development of gaps and bottleneck components of our study questionnaire.

Although the literature review was very instrumental in clarifying an appropriate approach to the process for setting an operational research agenda as well as identifying research gaps and needs, the country operational research dialogue committee set up to organise setting MOR agenda for Nigeria still considered it necessary to conduct further local-based survey to identify more indigenous gaps and needs peculiar to the Nigerian context. Thus, this study was designed to interview academia, malaria program implementers, and policymakers from the six geo-political regions in Nigeria to generate a full complement information required for MOR agenda setting.

Relevant documents reviewed in NMEP are listed (Additional file 1).

### Consultative process

#### Planning meetings

A planning committee for country operational research dialogue comprising head of thematic branches (case management and drug policy, monitoring and evaluation, advocacy communication and social mobilization, integrated vector management, programme management, procurement and supply chain management) of NMEP and other malaria control partners including NFELTP and led by the NMEP was formed. The committee was saddled with the development of a MOR agenda and planning of a national MOR stakeholders’ workshop to set the MOR agenda. Meanwhile, the NFELTP in collaboration with NMEP formed a MOR agenda setting preliminary survey task-team comprising NFELTP graduates and resident, NMEP programme officers and university researchers to conduct preliminary studies towards identification of operational research gaps, bottlenecks and needs for deliberation at the National MOR Stakeholders Workshop. The twelve-member task-team had an inaugural meeting on 8th October, 2016; thereafter held a seven-day brainstorming sessions, reviewed/surveyed literature, developed protocol and data collection tools, consulted stakeholders and designed framework for preliminary survey (Figure). The activities of the task-team span from October 2016 to March 2017.

### Primary data survey

#### Study design

A formative research consultative study design was used [9]. After an initial scoping review, primary data were collected from October to December 2016 using mixed methods - online survey, self-administered paper-based survey, key informant interviews with developmental partners, policy makers, malaria experts and programme managers. The questionnaire for online and self-administered paper-based survey was adapted from a previous study and revised to address of our study [9].

#### Study population

Inclusion criteria of researchers were active participation in malaria research in the last one decade. Global, national and local stakeholders were approached to participate in the study. These included officers in relevant units and divisions of Ministries of Health and their parastatals, NMEP research collaborators, research institutions such as National Institute of Medical Research, Academia (universities), Roll Back Malaria partners, non-governmental organisations, NFELTP and individual researchers (national and international). We included all contactable persons with self-identified expertise in malaria control and research in Nigeria, researchers in diaspora with published work from data collected in Nigeria and individuals in identified organisations and institutions working on malaria and have malaria related publications.

#### Sample size and sampling technique

All the relevant stakeholders that could be identified across six geopolitical zones of Nigeria and globally were surveyed. We included participants purposively based on their malaria research focus and involvement in malaria programme activities in Nigeria.

#### Data collection

Primary data were collected from October to December 2016 using mixed methods - online survey (*n* = 100), self-administered paper-based survey (*n* = 85) and key informant interviews (KII) with development partners, experts, policy makers (*n* = 40). In addition, a desk review with thematic heads of NMEP (*n* = 22) was held to generate list of gaps/bottlenecks, concerns and needs related to OR in their branches. The survey questionnaires and KII guide contained information on themes identified a priori and requested information on researchers and other stakeholders experience with MOR, thematic areas of research gaps, related research needs and their ranking (Additional files 2 and 3). The themes include (i) malaria prevention including chemoprevention, insecticides, long-lasting insecticidal nets, environmental management and vector behavior (ii) case management including diagnosis and treatment (iii) surveillance, monitoring and evaluation (iv) advocacy, communication and social mobilization including social behavioural change communication (v) programme management including funding, policy and coordination and (vi) procurement and supply chain management. Other information sought include cross cutting issues such as choice of MOR, how research findings were disseminated and to who, perceived importance of research agenda setting and what should be the content of a research agenda, suggestions relating to the process for research agenda setting and stakeholders to be included in malaria research agenda setting.

The data collection instruments were pre-tested among nine malaria stakeholders in Abuja-Nigeria who did not participate in the survey, prior to final use. The task-team members were trained as interviewers. They were paired to conduct the KII interviews which were audio-recorded and notes were taken. The online survey was administered using Survey Monkey®. Prospective respondents were invited via e-mail to complete the online questionnaires over a period of 5 weeks (21st October – 17th November, 2016). Reminders were sent at seven-day intervals. The online survey took the respondents 15–20 min to complete. The KII interviews were held at the convenience of the participants and in a secluded area in their offices or institutions.

### Desk review/situational analysis workshop

A two-day desk review/situational analysis workshop of progress in malaria operational research priorities by thematic areas was held with 22 participants including the National Coordinator of NMEP; thematic heads of branches and technical officers. The aims were to identify what has been achieved regarding MOR, the emerging OR issues in malaria control/elimination, determine the current NMEP OR gaps and needs and develop a plan to indicate milestone and key indicators of MOR.

### Data analysis and result writing workshop

The online and self-administered paper-based survey data entered in Microsoft excel were analysed using Statistical Package for Social Sciences version 20 [10]. A ten-day data analysis and result writing workshop was held. One qualitative and another quantitative data analysis expert led the data analysis workshop. The data were summarised using descriptive statistics. Thematic analysis procedures were used in presenting the KIIs data. The tape-recordings of the KIIs were transcribed and coded using ATLAS. ti version 7.5 [11]. Coding was done using an integrated approach which involved a combination of deductive and inductive coding procedures. The codes were appropriately linked to quotations and memos. Themes were derived based on patterns of results as reflected in codes, quotations and memos; and afterwards, were organised to reflect the interviewees’ major ideas, contrasting views and striking or salient points [12]. Research gaps were categorised under specific themes. The findings were explored further by consultation with relevant stakeholders using Delphi interview.

### Delphi interview

Delphi interviews were held with participants purposively selected out of those who participated in the survey (*n* = 8). This was conducted by face to face interview to further explore issues identified from survey and KII interviews, requiring further clarification and supporting/in-depth information. These issues include (i) appropriate source of funds for MOR (government or donors) as donors tend to dictate type of research/interventions and this militate against the conduct of MOR (ii) researchers are not obliged to share their research findings with government programmes if they are not funded by government (iii) policy conflict such as agenda of foreign funding agencies are different from the local MOR agenda and (iv) collaboration on MOR between NMEP and researchers. Content analysis of the transcribed Delphi-interviews was done. Further thematic analysis and regrouping of results were carried out. Research gaps were refined further and short listed.

## Results

Response and completion rates of online survey were 31.8% (100/314) and 48.0% (48/100), respectively. Response rate for self-administered paper-based survey was 85.0% (85/100). The outcomes, identified research gaps, bottlenecks and needs by thematic areas identified were made available to the NMEP towards a two-day national stakeholder workshop on malaria operation research agenda (held on 8–9 February, 2017) to offer data to formulate malaria research questions and develop a national MOR agenda. As part of dissemination process, manuscripts on findings of the KII and quantitative survey have been published [12, 13]. The outcome of the overall process is contained in “The National Malaria Operations Research Agenda (NMORA)” document [14]. Research institutions including private researchers, and malaria development partners in Nigeria and beyond are expected to implement the MOR questions within the life span of The NMORA document (2017–2020) to achieve the goal of malaria elimination [14].

## Discussion

To our knowledge, this is the first attempt at using a systematic approach comprising multilevel data collection methods to provide preliminary data for setting a national MOR. This was based on consultative design and use of mixed methods. We have described the processes involved in this paper. A similar consensus-based approach has been suggested earlier [15, 16] and used in other settings and initiatives for developing research agenda [9, 16]. However, our approach differs from that used by NMEP in previous years whereby only selected panel of experts participated in developing MOR agenda.

The approach described has an advantage of being led by NMEP, unlike reported from a previous study [17]. Having national malaria programmes take the lead in setting MOR agenda is crucial because of their intimate knowledge of the real situation and familiarity with the gaps and the bottleneck associated with malaria programme implementation [18]. This has a potential to foster implementation of local research on currently identified challenges using local solutions which, can be sustained and could encourage government ownership and building of local research capacity [19]. The leadership role of NMEP which fostered broad stakeholder engagement shows organisational and implementation readiness which Weiner emphasized [7]. Contrarily, MOR has been dictated by donors’ interests and do not result in sustainable solutions as they are imposed and often do not address local needs [19]. Moreover, our approach has provided a consensus-based comprehensive approach for developing MOR [14] for NMEP and presented a platform for strengthening linkage of NMEP and academic and training institutions. This is useful at a time when the NMEP aims to achieve pre-elimination and zero mortality. This political will is crucial for effective implementation of Nigeria national MOR document [14] having defined current knowledge gaps that can be addressed by the academia and developed appropriate strategies with robust stakeholder partnership [7, 8].

World Health Organisation emphasised the paramount need for innovative research that meets current specific priorities [3] and the need to continue to search for knowledge on more effective ways to implement current strategies to ensure adequate coverage of intervention strategies [20]. Our approach eliminated individual biases associated with interests of funders or malaria experts and adhered to principle of legitimacy and fairness [21]. Involvement of diverse range of stakeholders with different backgrounds across the six geopolitical zones of the country attests to its legitimacy. This robust approach in setting Nigeria MOR will help mitigate against non-implementation or fragmented implementation when major stakeholders are not involved [22]. The processes described in this paper have provided much needed information for setting updated agenda during the period of the NMSP.

The response rate recorded in our study, though low (31.8%), was higher than 8.4% reported elsewhere for online survey for research agenda setting [9]. Ali et al. reported a much higher response rate [57%] probably because participants were recruited using snow-ball sampling and this increased likelihood of response [23]. Our study was the first attempt at using a web-based approach for eliciting responses on setting a country-specific operational research agenda, this is novel in Nigeria and could have accounted for the low response rate. Low internet penetration or probable aversion to online survey could have contributed to this as well. Moreover, in our study, the online survey was complemented by self-administered paper-based survey, a replica of online survey questionnaire and this seemed to have provided saturated feedback on information on gaps and needs to MOR. Assuming we had offered incentives for participation, this could have increased participation rate in the survey as shown in previous studies [24, 25].

One limitation of this approach to setting MOR is that it did not include stakeholders at the health facility and community levels who are the end-users. Involvement of non-technical stakeholders in setting operational research agenda has been suggested [26]. To address this, the participants in the process of setting the MOR agenda had immense experience implementing MOR at these levels. Although the participants in this study were purposively selected based on their published research work and involvement in programme activities, majority were from southern part of the country. This could be a reflection of the extent of malaria research activities across the country.

## Conclusion

The structured and scientific approach for setting national MOR is a paradigm shift towards research agenda setting in Nigeria. This has an implication for researchers who intend to conduct research that can influence policy and practice. They are better guided as to the national MOR focus and their participation in the generation of the evidence-based information stands to stimulate their interest in MOR. This consensus-based stepwise approach will be useful for future plans for MOR agenda setting, and can be adopted for developing robust OR agenda for other public health initiatives in Nigeria and other settings.

## Additional files

Additional file 1: List of Reviewed Documents from the National Malaria Elimination Programme (available at www.nmcp.gov.ng/archives, accessed 2016 October 7). (DOCX 29 kb)

Additional file 2: Nigeria National Malaria Operational Research Agenda Online Survey questionnaire. (DOC 144 kb)

Additional file 3: Nigeria National Malaria Operational Research Agenda Key Informant Interview Guide. (PDF 44 kb)

## Abbreviations

KII: Key Informant Interview
MOR: Malaria Operational Research
NFELTP: Nigeria Field Epidemiology and Laboratory Training Programme
NMEP: National Malaria Elimination Programme
NMORA: National Malaria Operations Research Agenda
NMSP: National Malaria Strategic Plan
