# Supplementary material for: Setting a Nigeria national malaria operational research agenda: the process
Source: BMC Health Serv Res. 2018 Jun 18;18:459. doi: 10.1186/s12913-018-3224-5 (PMC6004659; doi:10.1186/s12913-018-3224-5)
Supplement: Supplementary file 1 — List of Reviewed Documents from the National Malaria Elimination Programme (available at www.nmcp.gov.ng/archives, accessed 2016 October 7). (DOCX 29 kb) [file 12913_2018_3224_MOESM1_ESM.docx]

**Additional file 1:**

**List of Reviewed Documents from the National Malaria Elimination Programme (available at** [www.nmcp.gov.ng/archives](http://www.nmcp.gov.ng/archives)**, accessed 2016 October 7)**

National Malaria Strategic Plan 2014 – 2020

Report on data collection and synthesis for National Malaria Control Programme Operational Research April/August 2010

National Malaria Policy, 2014

National Strategy and Guidelines for prevention and control of malaria in pregnancy, Second Edition, February 2014

National Guidelines for Malaria Diagnosis and Treatment, Third Edition, May 2015

Report of the Country Dialogue on Malaria Operations Research, December, 2014.

Policy Framework for The Development and Implementation of Integrated Vector Management In Nigeria, July 2005

A Framework for Malaria Procurement and Supply Chain Management in Nigeria, February 2014

Guidelines for Malaria Advocacy Communication and social mobilization programme 2014
